# Metabolic Reprogramming and Inflammatory Response Induced by D-Lactate in Bovine Fibroblast-Like Synoviocytes Depends on HIF-1 Activity

**DOI:** 10.3389/fvets.2021.625347

**Published:** 2021-03-16

**Authors:** John Quiroga, Pablo Alarcón, Carolina Manosalva, Stefanie Teuber, Anja Taubert, Carlos Hermosilla, María Angélica Hidalgo, María Daniella Carretta, Rafael Agustín Burgos

**Affiliations:** ^1^Laboratory of Inflammation Pharmacology, Faculty of Veterinary Sciences, Institute of Pharmacology and Morphophysiology, Universidad Austral de Chile, Valdivia, Chile; ^2^Laboratory of Immunometabolism, Faculty of Veterinary Sciences, Institute of Pharmacology and Morphophysiology, Universidad Austral de Chile, Valdivia, Chile; ^3^Faculty of Sciences, Institute of Pharmacy, Universidad Austral de Chile, Valdivia, Chile; ^4^Biomedical Research Center Seltersberg, Institute of Parasitology, Justus Liebig University Giessen, Giessen, Germany

**Keywords:** bovine fibroblast-like synoviocyte, D-lactate, metabolic reprogramming, inflammation, hypoxia inducible factor 1

## Abstract

Acute ruminal acidosis (ARA) occurs after an excessive intake of rapidly fermentable carbohydrates and is characterized by the overproduction of D-lactate in the rumen that reaches the bloodstream. Lameness presentation, one of the primary consequences of ARA in cattle, is associated with the occurrence of laminitis and aseptic polysynovitis. Fibroblast-like synoviocytes (FLS) are predominant cells of synovia and play a key role in the pathophysiology of joint diseases, thus increasing the chances of the release of pro-inflammatory cytokines. Increased D-lactate levels and disturbances in the metabolism of carbohydrates, pyruvates, and amino acids are observed in the synovial fluid of heifers with ARA-related polysynovitis prior to neutrophil infiltration, suggesting an early involvement of metabolic disturbances in joint inflammation. We hypothesized that D-lactate induces metabolic reprogramming, along with an inflammatory response, in bovine exposed FLS. Gas chromatography-mass spectrometry (GC-MS)-based metabolomics revealed that D-lactate disrupts the metabolism of bovine FLS, mainly enhancing glycolysis and gluconeogenesis, pyruvate metabolism, and galactose metabolism. The reverse-transcription quantitative PCR (RT-qPCR) analysis revealed an increased expression of metabolic-related genes, including hypoxia-inducible factor 1 (HIF-1)α, glucose transporter 1 (Glut-1), L-lactate dehydrogenase subunit A (L-LDHA), and pyruvate dehydrogenase kinase 1 (PDK-1). Along with metabolic disturbances, D-lactate also induced an overexpression and the secretion of IL-6. Furthermore, the inhibition of HIF-1, PI3K/Akt, and NF-κB reduced the expression of IL-6 and metabolic-related genes. The results of this study reveal a potential role for D-lactate in bFLS metabolic reprogramming and support a close relationship between inflammation and metabolism in cattle.

## Introduction

Acute ruminal acidosis (ARA) is a well-known metabolic syndrome in cattle caused by excessive intake of rapidly fermentable carbohydrates, which alters ruminal microbiota composition (1, 2). As such, the proliferation of lactate-producing bacteria, primarily *Streptococcus bovis* and *Lactobacillus* spp., leads to excessive production of D-lactate and L-lactate and a consequent drop in ruminal pH (1, 3). During ARA episodes, sufficient L-lactate is absorbed into the forestomach and from more distal portions of the digestive tract into the bloodstream (4, 5). L-Lactate is rapidly oxidized to pyruvate by L-lactate dehydrogenase, primarily in cardiac tissues and hepatic tissues (3). Nevertheless, D-lactate can be metabolized, albeit less efficiently, by a mitochondria-derived enzyme, D-2-hydroxy acid dehydrogenase (D-LDH dehydrogenase) (6, 7), in such a way that it accumulates in the blood system at a concentration greater than 5 mM, thereby causing D-lactic acidosis (4, 5). D-lactic acidosis impairs animal welfare and the economic performance of cattle, as it affects feed intake and ruminal digestion, causing rumen mucosa damage (ruminitis), liver abscesses, diarrhea, inflammation, and lameness (2, 3, 8). Distension of the tarsocrural joints in dairy heifers with ARA has been observed (9–12) and is characterized by generalized sterile neutrophilic polysynovitis (9). This acute joint reaction is considered to be a part of the clinical complex interpreted as acute laminitis, and although the clinical consequence is still unclear, it most likely contributes to claw pain and lameness (9–11).

Fibroblast-like synoviocytes (FLS) are the predominant cell types of synovial intima and assure the structural and physiological dynamic integrity of diarthrodial joints, controlling the composition of synovial fluids and the extracellular matrix of the joint lining (13). Moreover, FLS play a central role in defining and maintaining an inflammatory environment during joint diseases (13, 14). Furthermore, activated FLS exhibit metabolic disturbances and produce mediators that can induce angiogenesis, cell growth, leukocyte recruitment, and immune cell activation (14–16).

High levels of D-lactate and significant changes in the metabolism of carbohydrates, pyruvates, and amino acids have been detected in the synovial fluid of heifers with polysynovitis associated with ARA prior to subsequent neutrophil infiltration, suggesting an extremely early involvement of metabolic disturbances in bovine joint inflammation (17). Similarly, increased levels of lactate and high rates of glucose consumption have been detected in human joints with aseptic inflammation, which is primarily attributed to the activation of hypoxia-inducible factor 1 (HIF-1) (14, 18). Lactate has also been identified as a pro-inflammatory agent in FLS and macrophages, inducing prostaglandin E_2_ (PGE_2_) release into the medium (19). Furthermore, lactate induces the secretion of interleukin (IL)-6, tumoral necrosis factor-α (TNF-α), and IL-1β in stimulated chondrocytes (20) and increased the production of PGE_2_ and the activity of gluconeogenic in lactate-exposed monocytes (21) through a HIF-1-dependent mechanism, suggesting that the lactate-induced inflammatory response is dependent on these co-induced metabolic adaptations. Additionally, lactate has been identified to be responsible for TNF-α-induced IL-6 production in human rheumatoid FLS through the activation of nuclear factor kappa B (NF-κB) (22).

Based on the abovementioned findings, we hypothesized that D-lactate might be able to induce metabolic disturbances and inflammatory responses in bovine FLS (bFLS). The present study demonstrates that D-lactate caused significant metabolic changes, which primarily involved the metabolism of carbohydrates and amino acids. In association with this metabolic reprogramming, we observed that D-lactate induced the mRNA expression of relevant pro-inflammatory genes and metabolic genes in a phosphatidylinositol 3-kinase (PI3K)/protein kinase B (Akt)-dependent manner, as well as in a HIF-1/NF-κB-dependent manner.

## Materials and Methods

### bFLS Cell Culture

Bovine FLS (#CDD-B-2910, Articular Engineering, Northbrook, IL, USA) were cultured in sterile 25 cm^2^ plastic tissue culture flasks (#70025, SPL Life Sciences, Pocheon-si, Korea) with Dulbecco's Modified Eagle/Ham's F-12 (DMEM/F-12; #12400016, Gibco, Thermo Fischer Scientific, Waltham, MA, USA) supplemented with 10% fetal bovine serum (FBS; #S1810, Biowest, Nuaillé, France) at 37°C under an atmosphere with 5% CO_2_. Cell linage was confirmed by the presence of Vimentin and the absence of CD14, according to Manosalva et al. (23). During passages 3–6, bFLS were cultured in DMEM/F-12 supplemented with 10% FBS in sterile 21.5 cm^2^ plastic tissue culture plates (#20060, SPL Life Sciences, Pocheon-si, Korea) for metabolomics and lactate and immunoblot analyses, while sterile 6-well plates (#31006, SPL Life Sciences, Pocheon-si, Korea) were used for reverse transcription-quantitative PCR (RT-qPCR) and ELISA analysis.

### Experimental Design

Inhibitory assays were performed at 37°C and under 5% CO_2_ for 30 min using the following pharmacological inhibitors: (a) 40 μM YC-1 (#sc-202856, Santa Cruz Biotechnology, Santa Cruz, CA, USA) to inhibit the HIF-1 activity; (b) 10 μM BAY 11-7082 (#10010266, Cayman Chemicals, Ann Arbor, MI, USA) to inhibit the NF-κB activity; and (c) 10 μM LY294002 (#V1201, Promega, Madison, WI, USA) to inhibit the PI3K/Akt signaling pathway. DMSO (0.1%) was used as vehicle control. bFLS were stimulated with 5 mM D-lactate (#L0625, Sigma-Aldrich, St. Louis, MO, USA) or 100 ng/ml bovine TNF-α (bTNF-α; #RBOTNFAI, Thermo Fisher Scientific, Waltham, MA, USA) at 37°C and under 5% CO_2_ for 1 h for metabolomics and lactate analysis and 6 h for immunoblot analysis and RT-qPCR and ELISA experiments. Water was used as vehicle control. Furthermore, for immunoblot analysis, hypoxic conditions (1% O_2_, 94% N_2_, 5% CO_2_) were achieved using a hypoxia incubator chamber (#27310, Stemcell Technologies, Vancouver, Canada). For HIF-1α stabilization control, 300 μM cobalt chloride (CoCl_2_) was added to bFLS under normoxic conditions.

### Sample Preparation for Gas Chromatography-Mass Spectrometry (GC-MS) Metabolomics

Metabolites from bFLS were extracted with 1 ml/sample of extraction buffer (37.5% vol/vol HPLC-grade acetonitrile; 37.5% vol/vol HPLC-grade isopropanol; 25% vol/vol HPLC-grade water) containing 1 mM ribitol (#A5502, Sigma-Aldrich, St. Louis, MO, USA) as an internal standard. Samples were vortexed for 2 min and then centrifuged at 14,000 × g at 4°C for 2 min. Later, 450 μl-supernatant from each sample was dried in a SpeedVac concentrator (Savant^®^ SPD131DDA, Thermo Fisher Scientific, Waltham, MA, USA) at 45°C for 90 min under 1.5 atm of pressure. Once dried, 450 μl/sample of wash buffer (50% vol/vol HPLC-grade acetonitrile; 50% vol/vol HPLC-grade water) was added, which were then vortexed and centrifuged at 14,000 × g at 4 °C for 2 min; then, the supernatants were evaporated to dryness in a SpeedVac concentrator at 45°C for 90 min under 1.5 atm of pressure. As retention index markers, 2 μl of a fatty acid methyl ester (FAME) standard mixture C8-C30 (#400505, Fiehn GC/MS Metabolomics Standards Kit, Agilent Technologies, Santa Clara, CA, USA) was utilized. Additionally, 10 μl methoxyamine hydrochloride/pyridine (20 mg/ml; #226904, Sigma-Aldrich, #107463, Merck KGaA, Darmstadt, Germany) was added to each of the samples and incubated at 30°C for 90 min under shaking conditions. Subsequently, 90 μl N-methyl-N-(trimethylsilyl)-trifluoroacetamide (MSTFA) with 1% trimethylchlorosilane (TMCS) derivatization agent (#TS-48915, Thermo Fisher Scientific, Pierce Biotechnology, Rockford, IL) was added, and samples were incubated at 37°C for 30 min under shaking conditions. Finally, samples were transferred to 250-μl glass vial inserts (#5181-8872, Agilent Technologies) in 2-ml glass vials with screw caps (#8010-0543, Agilent Technologies) for analysis.

### Metabolomics by GC-MS

Samples were injected in an Agilent 7890B GC system coupled to an electron impact ionization mode 5977 A Mass Selective Detector system (Agilent Technologies, Palo Alto, CA, USA), using an Agilent 7693 Series Autosampler (Agilent Technologies, Palo Alto, CA, USA). Derivatized samples (2 μl) were injected in the splitless injector mode on a 30 m × 0.25 mm × 0.25 μm DB-5 column (Agilent Technologies, Palo Alto, CA, USA). Temperature of the injector port was maintained at 250°C, and the flow rate of helium carrier gas was set up at 1 ml/min with an initial oven temperature of 60°C. Then, the oven temperature was increased at 10°C/min until it reached 325°C, with a final running time of 37.5 min. After a 5.9 min solvent delay, full spectra (50–600 m/z; 1.7 scans/s) with a digital scan rate of 20 Hz, with MS ion source temperature of 250°C and quadrupole temperature of 150°C, was acquired. All samples were analyzed within 24 h after derivatization. To calculate the Fiehn retention index of metabolites, retention times were obtained by injecting a FAME standard mixture C8-C30 (#400505, Fiehn GC/MS Metabolomics Standards Kit, Agilent Technologies, Santa Clara, CA, USA).

Before carrying out data analysis, raw MS data (.D files) were transformed into the Analysis Base File (.ABF) format using the Reifycs ABF Converter (Reifycs Inc., Tokyo, Japan). Metabolite identification was performed following the methods described by Fiehn (24). Briefly, peak detection, deconvolution, and peak alignment were performed using MSDIAL 2.83 (RIKEN Center for Sustainable Resource Science: Metabolome Informatics Research Team. Yokohama, Japan) to process the total ion chromatogram and the electron ionization-MS (EI-MS) spectra of each GC peak. The resulting mass spectrum of the trimethylsilyated metabolites was identified, and the deconvoluted peaks were matched against mass spectral libraries imported by the National Institute of Standards and Technology (NIST) MSP format. Library matches were ranked against experimental data based on the total retention index and mass spectral similarity across all batch samples. The Fiehn retention index based on FAME was used. Identification of metabolites was performed by matching the EI-MS spectra with those of the reference compounds from the NIST or Fiehn libraries. For analysis, the retention index tolerance of 2,000, a EI similarity cutoff of 65%, an identification score cutoff of 70%, a mass scale tolerance of 0.5 Da, and the retention time tolerance of 0.5 min were used.

### Quantification of Intracellular D-Lactate and L-Lactate by High-Performance Liquid Chromatography (HPLC)

Lactate stereoisomers from bFLS were extracted with 1 ml/sample of extraction buffer (37.5% vol/vol HPLC-grade acetonitrile; 37.5% vol/vol HPLC-grade isopropanol; 25% vol/vol HPLC-grade water) by vortexing for 2 min and performing centrifugation at 14,000 × g at 4°C for 2 min. Next, 450 μl-supernatant from each sample was dried in a SpeedVac concentrator (Savant^®^ SPD131DDA, Thermo Fisher Scientific, Waltham, MA, USA) at 45°C for 90 min under 1.5 atm of pressure. Once dried, the samples were resuspended in 250 μl mobile phase (1 mM CuSO_4_) and centrifuged at 21,000 × g at 4°C for 10 min. Finally, 200 μl-aliquots of the supernatants were used for D- and L-lactate quantification. For the calibration curves, 2–400 μM of D- and L-lactate standards were used. Twenty-microliter aliquots of samples were analyzed by HPLC using an Astec CLC-D cationic exchange column (15 cm × 4.6 mm; Sigma-Aldrich, St. Louis, MO, USA) at a flow rate of 1 ml/min at 30°C. The detection wavelength was set at 254 nm (25) using LaChrom Elite HPLC Diode Array Detector (VWR Hitachi, Radnor, PA, USA).

### Western Blot Analysis

Total proteins were extracted with 2 × Laemmli sample buffer (0.125 M Tris-HCl, pH 6.8; 4% SDS; 20% glycerol; 10% β-mercaptoethanol; 0.004% bromphenol blue). Total proteins were separated by electrophoresis using 7.5% SDS-PAGE gels and transferred electrophoretically into nitrocellulose membranes. After blocking with 5% skim milk in TBS-T (20 mM Tris-HCl, pH 7.5; 137 mM NaCl; 0.1% Tween 20), the membranes were incubated overnight with an anti-HIF-1α monoclonal antibody (H1alpha67) (#MA1-16504; Invitrogen, Thermo Fischer Scientific) and an anti-β-actin [horseradish peroxidase (HRP)] antibody (#sc-47778; Santa Cruz Biotechnology) at 4°C. Finally, the membranes were incubated with a HRP-conjugated anti-mouse IgG antibody (#115-035-003; Jackson Immunoresearch, West Grove, PA, USA). Specific bands were visualized using the Odyssey Fc Dual-Mode Imaging System (LI-COR Biosciences, Lincoln, NE, USA), and its intensity was quantified using the Image Studio Lite v5.2 software (LI-COR Biosciences).

### RT-qPCR Analysis of Inflammatory and Metabolic Genes

Total RNA of bFLS was extracted with an E.Z.N.A. Total RNA Kit I (#R6834-01, Omega Bio-Tek, Norcross, GA, USA) following the instructions of the manufacturer. To remove genomic DNA, the extracted RNA was treated using a Turbo DNase-Free^®^ kit (#AM1907, Ambion™, Thermo Fischer Scientific, Waltham, MA, USA). For cDNA synthesis, 300 ng of total RNA was reverse transcribed using M-MLV reverse transcriptase (#M5313, Promega, Madison, WI, USA) according to the protocol of the manufacturer. RT-qPCR assays were performed using Takyon™ Rox SYBR^®^ MasterMix (#UF-RSMT-B0701, Eurogentec, Seraing, Belgium), and the primers are indicated in Table 1. RT-qPCR was performed in a StepOne Plus Real-Time PCR System (Applied Biosystems™, Thermo Fisher Scientific, Waltham, MA, USA) using the following cycling conditions: 1 cycle at 95°C for 10 min, followed by 40 cycles at 95°C for 30 s, 60°C for 30 s (annealing), and 72°C for 30 s (extension). The changes in expression were calculated using the 2^−(ΔΔCt)^ method, according to Livak and Schmittgen (26), using StepOne™ v2.3 (Applied Biosystems™, Thermo Fisher Scientific). For normalization, the 40S ribosomal protein S9 (RPS9) as a housekeeping gene and as unstimulated cells as a reference sample were used.

**Table 1:** Target genes, forward and reverse primer (5′-3′) sequences, amplicon size (pb), regression coefficient (R^2^) value, slope, and amplification efficiency (%).

**Gene**	**Forward primer, 5′-3′**	**Reverse primer, 5′-3′**	**Size (bp)**	***R*^2^**	**Slope**	**Efficiency (%)**
*rps9*	GCTGACGCTGGATGAGAAAGACCC	ATCCAGCACCCCGATACGGACG	85	0.995	−3.612	89.164
*il-6*	ACTGGCAGAAAATAAGCTGAATCTTC	TGATCAAGCAAATCGCCTGAT	89	0.998	−3.523	92.239
*il-8*	ATGACTTCCAAGCTGGCTGTTG	TTGATAAATTTGGGGTGGAAAG	149	0.998	−2.920	120.040
*hif-1α*	GGAGTTGGACCTCTGCGATT	GAGGGGAGAAAAGGCACGTC	102	0.995	−3.267	102.331
*glut-1*	GCGGACCCTACGTCTTCATC	GGCCTTTTGTCTCGGGAACT	87	0.999	−3.304	100.749
*pdk-1*	CTCATCGGAAACACGTCGGA	TCACACAGACGCCTAGCATT	91	0.996	−3.484	93.643
*l-ldha*	AGGCCTGAGAAGTCGGAGTG	GGAACCTGTCCTACCTGCC	118	0.983	−3.512	92.633

*rps9, 40S ribosomal protein S9; il-6, interleukin 6; il-8: interleukin 8; hif-1α, hypoxia inducible factor-1 subunit alpha; glut-1, solute carrier family 2 (facilitated glucose transporter), member 1; pdk-1, pyruvate dehydrogenase kinase 1; l-ldha, L-lactate dehydrogenase subunit A; bp, base pairs*.

### IL-6 and IL-8 Quantification by ELISA

After D-lactate stimulation for qPCR assay, conditioned media were centrifugated at 500 × g for 5 min and thereafter used to estimate the concentration of cytokines by using bovine IL-6 (#ESS0029, Thermo Fisher Scientific) and IL-8 (#3114-1A-6, Mabtech, Nacka, Sweden) ELISA kits, according to instructions of the manufacturer. Briefly, the capture antibody was incubated overnight, and wells were then blocked for 1 h (4% BSA, 5% sucrose in PBS). Subsequently, 100 μl of the sample was added and incubated for 1–2 h. After two washes, the detection antibody was incubated for 1 h. Plates were washed twice and streptavidin was added and incubated again for another 0.5–1 h. Finally, the tetramethylbenzidine (TMB) substrate solution or p-nitrophenyl phosphate (pNPP) was added and incubated for 20–30 min in the dark. For IL-6 ELISA kit, the reaction was stopped with 0.16 M H_2_SO_4_. All procedures were performed at room temperature. Plates were analyzed at 450 and 550 nm for the IL-6 ELISA kit and at 405 nm for the IL-8 ELISA kit using an automatic Varioskan Flash Reader (Thermo Fischer Scientific, Waltham, MA, USA).

### Statistical Analyses

For metabolomic analysis, all multivariate analyses were statistically analyzed using MetaboAnalyst v4.0 (Xia Lab, McGill University, Canada; http://www.metaboanalyst.ca) according to previously published protocols (27). Metabolites which were more than 50% below the detection limit or with at least 50% missing values were excluded from the analysis. Metabolite concentrations were normalized using ribitol as an internal standard, and to obtain a Gaussian distribution, logarithmic transformation and auto scaling were performed before the statistical analysis (27). The partial least squares-discriminating analysis (PLS-DA) and variable importance in projection (VIP) scores were determined. The PLS-DA model was estimated by cross-validation and permutation tests, as the sum of squares captured by the model (R2) > 0.9 and *p*-value = 0.0295 (59/2000), respectively. Heat maps were represented by Euclidean distance measure and Ward's clustering algorithm. Metabolites exhibiting significantly different levels (*p* < 0.05) by the Mann–Whitney test were considered for pathway topology analyses. *Bos taurus* (cow) was used as the model organism. Pathway topology analysis was performed using the *B. taurus* pathway library and a hypergeometric test was used for overrepresentation analysis. To identify potential metabolomic pathways, the Kyoto Encyclopedia of Genes and Genomes (KEGG; http://www.genome.jp/kegg) and the Bovine Metabolome Database (http://www.cowmetdb.ca) were used. For other experimental settings, data are presented as means ± SEM. For comparisons between two treatments, the Mann–Whitney test was applied. In addition, for comparisons between three or more groups, after variance homoscedasticity evaluation, the one-way ANOVA followed by Fisher's least significant difference test, or the Kruskal–Wallis test followed by Dunn's test, was applied as the appropriate method. Values of *p* < 0.05 were considered significant. PRISM v8.4.2 (GraphPad, San Diego, CA, USA) was used for statistical analyses.

## Results

### Metabolome Overview of Untreated- and D-Lactate-Treated bFLS

A total of 1,306 unique *m/z* values with retention indices were integrated after GC-MS analysis of bFLS, including internal standards. After deconvolution and alignment, 93 metabolites were identified and classified according to chemical classes (Supplementary Figure 1A). Metabolites were identified by the Fiehn retention index according to the FAME standard and the Fiehn library. The chemical structure of the derivative product was also used for metabolite identification. Carbohydrates and their metabolites were the primary compounds detected in bFLS (Supplementary Figure 1A), with 30 (32.6%) carbohydrates identified, including arabinose, fructose, galactitol, gluconic acid, glucose, glucose-1-phosphate, glucose-6-phosphate, glycerol, hexose, lactose, mannitol, mannose, melibiose, N-acetyl-D-hexosamine, sucrose, and UDP-N-acetylglucosamine. Amino acids and their derivatives were the second most important metabolites detected in the study (Supplementary Figure 1A), with 27 (29.3%) compounds identified, including 21 amino acids, such as alanine, aspartate, cysteine, glutamate, glycine, leucine, methionine, phenylalanine, proline, serine, threonine, tyrosine, and valine. Additionally, six amino acid derivatives, including 3-aminoisobutyric acid, ethanolamine, oxoproline, and putrescine, were detected. Seventeen (18.5%) lipidic compounds were identified, including arachidic acid, arachidonic acid, heptadecanoic acid, lauric acid, linoleic acid, myristic acid, oleic acid, palmitic acid, pentadecanoic acid, and stearic acid (Supplementary Figure 1A). Nine (9.7%) organic acids, including citric acid, fumaric acid, lactic acid, pyruvic acid, and succinic acid, were detected (Supplementary Figure 1A). One (1.1%) nucleoside was identified, corresponding to uracil (Supplementary Figure 1A). Other organic compounds, including 2.6-di-tert-butylphenol, methylamine, phosphate, and isothreonic acid, were also identified (Supplementary Figure 1A). Based on their relative abundances, hexose, ethanolamine, and mannitol were the three most predominant metabolites detected in bFLS, with other predominant metabolites including phosphate, myristic acid, leucine, myo-inositol, oxoproline, 1.2-anhydro-myo-inositol, and stearic acid (Supplementary Figure 1B). Detected metabolites with the lowest levels in bFLS were 2.6-di-tert-butylphenol, norleucine, fructose, inositol-4-monophosphate, arabinose, glucose-6-phosphate, tyrosine, palmitic acid, methylamine, and linoleic acid (Supplementary Figure 1C).

### D-Lactate Induces Metabolomic Changes in Exposed bFLS

**Figure 1:**
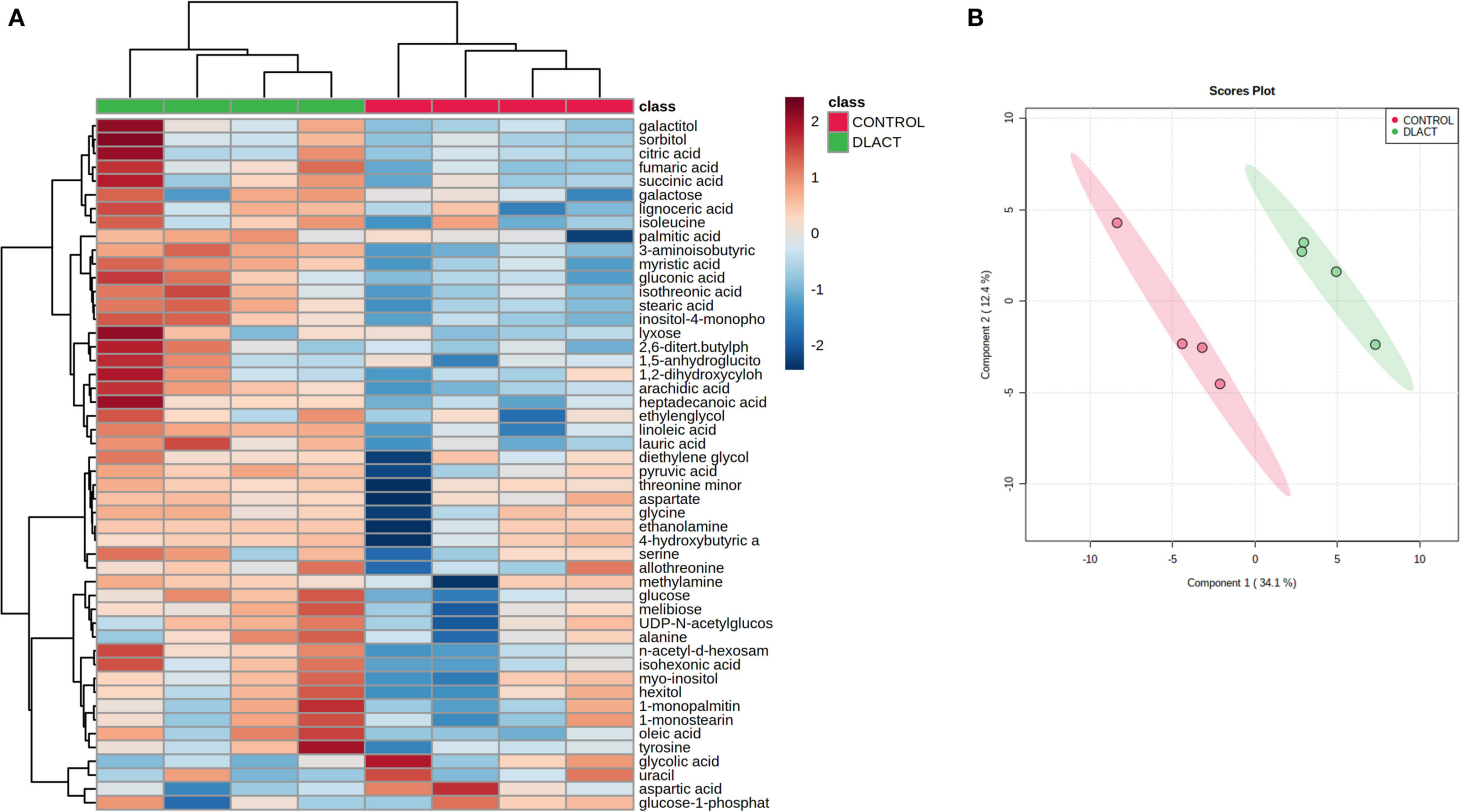
Metabolomic profile of bovine fibroblast-like synoviocytes (bFLS) after D-lactate treatment. **(A)** Hierarchical clustering analysis of 50 metabolites from the control untreated and 5 mM D-lactate-stimulated bFLS. The red and blue colors indicate that the metabolite level is increased and decreased compared to the mean metabolite relative abundance, respectively. Each column represents a sample clustered according to treatment, and each row represents an individual metabolite. *n* = 4. **(B)** The partial least squares-discriminant analysis (PLS-DA) score plot based on metabolomic analysis of D-lactate stimulated (green) and control (red) bFLS. The explained variances of the selected components are shown in brackets. *n* = 4.

**Figure 2:**
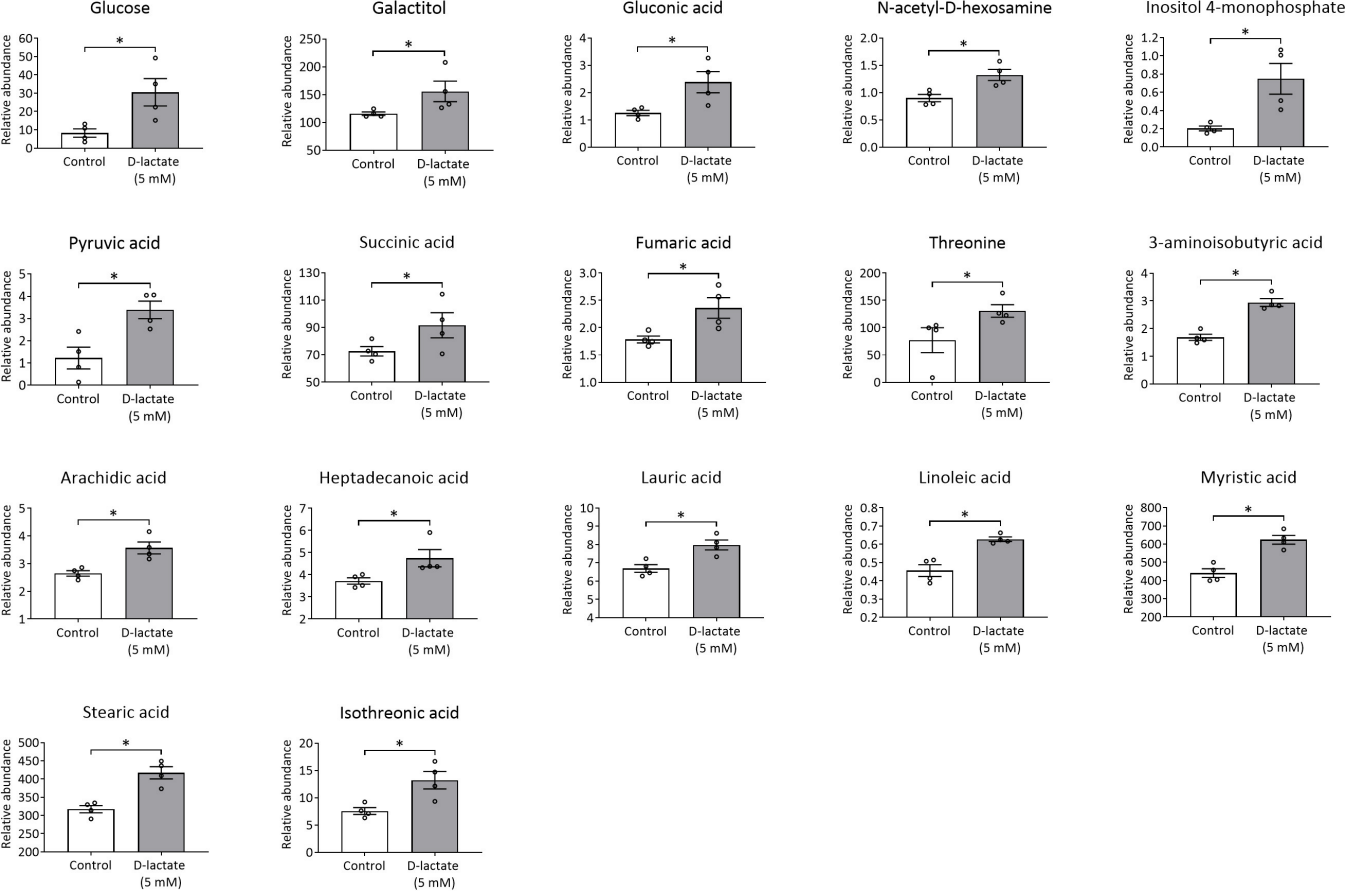
D-Lactate alters the intracellular concentration of several metabolites. The concentrations of metabolites significantly altered after 1 h stimulation with 5 mM D-lactate are expressed as relative abundance with respect to ribitol. Each bar represents the mean ± SEM. Each point represents an independent experiment, *n* = 4. *p < 0.05.

To evaluate the metabolic changes induced by D-lactate in exposed bFLS, we constructed a heatmap considering 50 metabolites with the lowest *p*-values, as determined by ANOVA. A distinctive hierarchical separation between control and D-lactate-treated bFLS was detected (Figure 1A). Furthermore, the PLS-DA showed a noticeable separation associated with D-lactate treatment, as axes 1 and 2 accounted for 34.1 and 12.4% of the total variation, respectively (Figure 1B). In this analysis, nine metabolites (3-aminoisobutyric acid, myristic acid, stearic acid, inositol-4-monophosphate, linoleic acid, arachidic acid, lauric acid, isothreonic acid, and N-acetyl-D-hexosamine) with the highest VIP scores (>1.6) contributed most significantly to the detected separation (Supplementary Figure 2). After univariate analysis, we observed 17 metabolites which were significantly altered by D-lactate treatment (Figure 2), with markedly increased levels of glucose (3.7-fold), inositol-4-monophosphate (3.7-fold), and pyruvic acid (2.8-fold). Gluconic acid, threonine, isothreonic acid, and 3-aminoisobutyric acid levels were also markedly increased (>1.7-fold) after stimulation with D-lactate. Finally, the treatment moderately (>1.3-fold) increased the levels of fumaric acid, galactitol, N-acetyl-D-hexosamine, arachidic acid, linoleic acid, myristic acid, and stearic acid, while a slight (>1.2-fold) increase in succinic acid, heptadecanoic acid, and lauric acid levels was observed (Figure 2). Overall, these results suggest that D-lactate induces significant changes in the metabolome of exposed bFLS.

**Figure 3:**
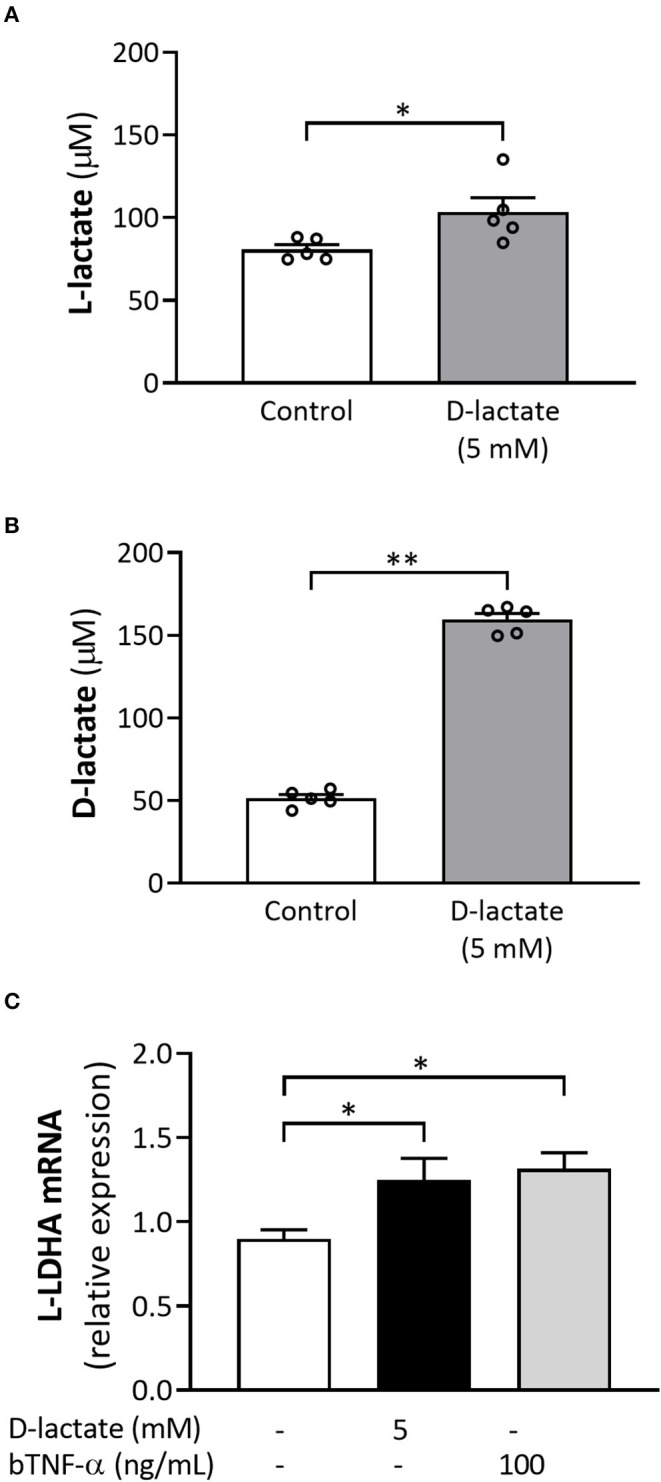
D-Lactate treatment increases the intracellular levels of both lactate stereoisomers as well as the L-lactate dehydrogenase subunit A (L-LDHA) expression. bFLS were treated with 5 mM D-lactate for 1 h. **(A)** L-lactate and **(B)** D-lactate were quantified at the intracellular level by high-performance liquid chromatography (HPLC). Each bar represents the mean ± SEM. Each point represents an independent experiment, *n* = 5. **(C)** The relative mRNA expression of L-LDHA was assessed by RT-qPCR. Bovine tumore necrosis factor-α (TNF-α) was used as the positive control. Each bar represents the mean ± SEM, *n* = 5. *p < 0.05; **p < 0.01.

Additionally, intracellular levels of L-lactate were quantified by HPLC in control and 5 mM D-lactate-treated bFLS. L-lactate concentrations were significantly higher in bFLS stimulated with D-lactate compared to control (Figure 3A). Intracellular D-lactate levels were also quantified by HPLC, which were three times greater than those observed in the bFLS control group (Figure 3B). These results show that intracellular levels of D-lactate increased after stimulation, which was also associated with an increase in intracellular production of L-lactate and LDHA expression (Figure 3C).

### D-Lactate Modifies Intracellular Metabolic Pathways in bFLS

**Figure 4:**
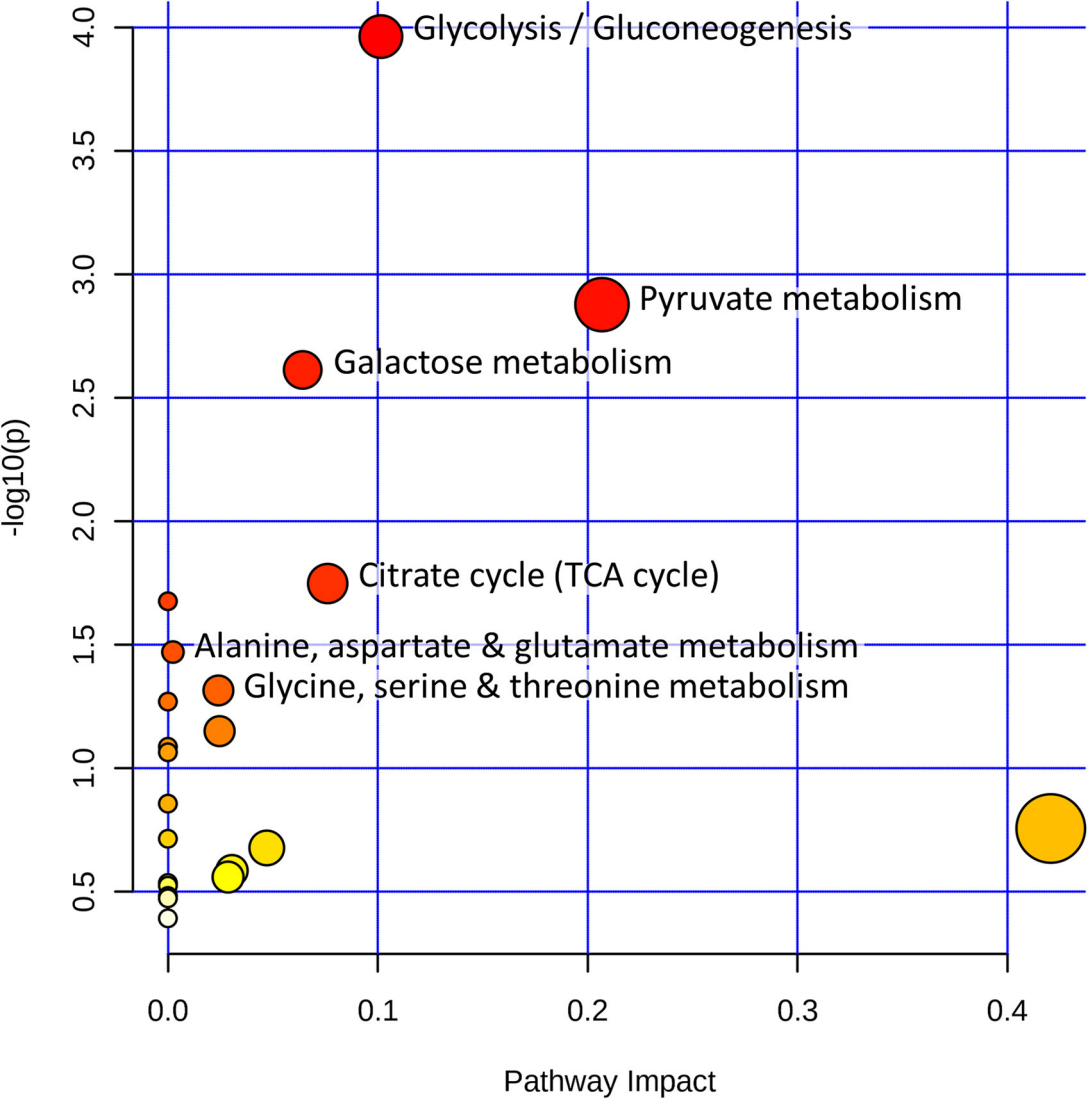
D-Lactate reprograms bFLS metabolism. bFLS were stimulated with 5 mM D-lactate for 1 h. Metabolic pathway analysis was performed using MetaboAnalyst v4.0 based on the Kyoto Encyclopedia of Genes and Genomes (KEGG) using all metabolites significantly altered after D-lactate treatment. All matched pathways are shown as circles. The color of the circles is based on *p*-values from pathway enrichment analysis, where darker colors indicate more significant metabolites changes in the corresponding pathway. The size of the circles represents the pathway impact score. The most impacted pathways with high statistical significance (*p* < 0.05) are labeled. *n* = 4.

We performed a metabolic pathway analysis with MetaboAnalyst v4.0 using a hypergeometric test for overrepresentation analysis of all significantly modified metabolites after D-lactate stimulation (Figure 4). The metabolic pathways that were most significantly modified and had a higher impact value were “glycolysis/gluconeogenesis;” “pyruvate metabolism;” “galactose metabolism;” “citrate cycle (TCA cycle);” “alanine, aspartate, and glutamate metabolism;” and “glycine, serine, and threonine metabolism” (Figure 4). These results showed that carbohydrate and amino acid metabolism were the primary metabolic pathways disturbed in bFLS after D-lactate treatment.

### D-Lactate Increases Expression of IL-6, HIF-1α, Glut-1, and PDK-1 in bFLS

**Figure 5:**
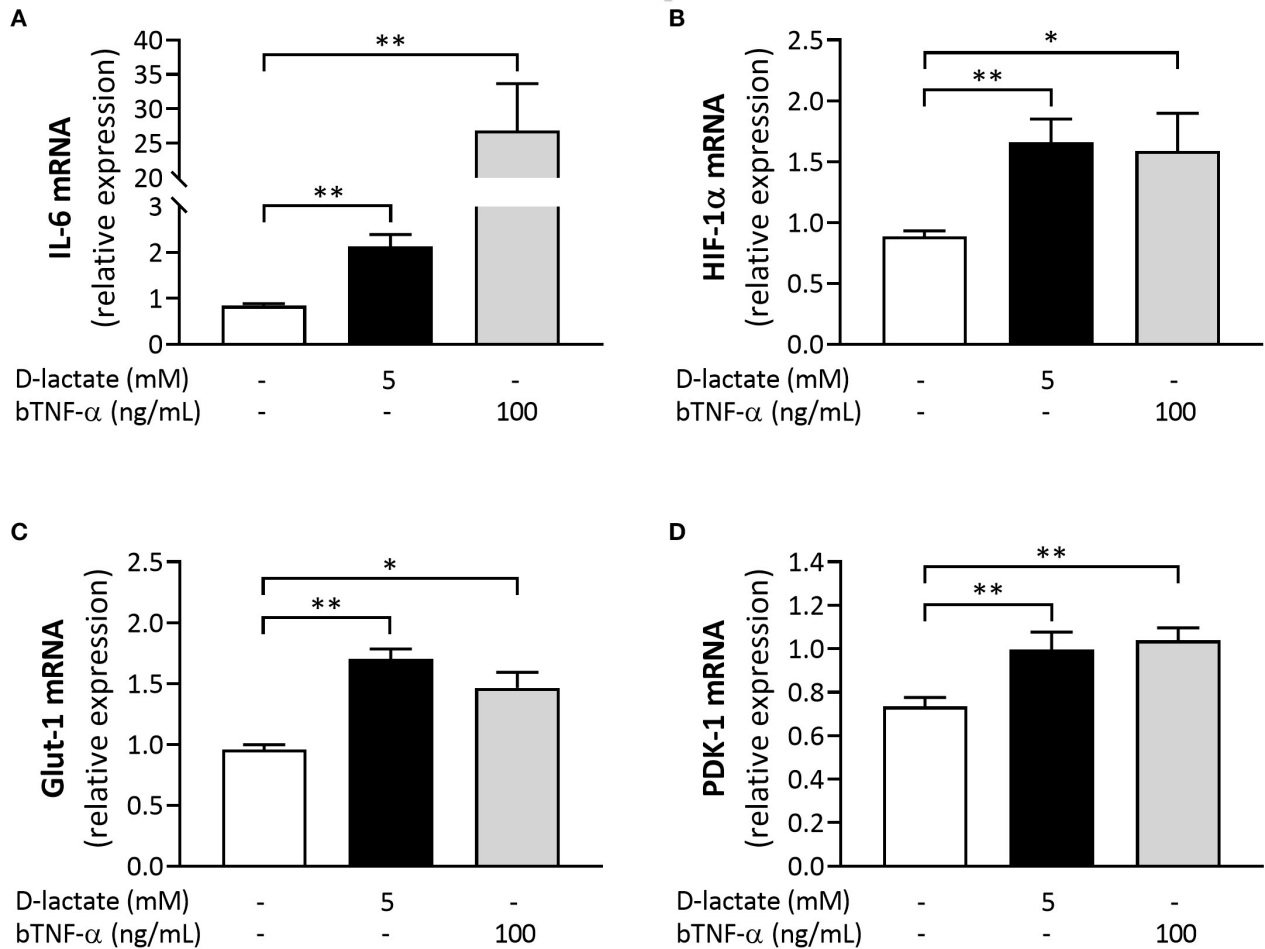
D-Lactate increases the expression of inflammation and metabolism-associated genes in bFLS. Relative mRNA expression of **(A)** IL-6, **(B)** hypoxia-inducible factor-1 subunit α (HIF-1α), **(C)** glucose transporter 1 (Glut-1), and **(D)** pyruvate dehydrogenase kinase 1 (PDK-1) in bFLS stimulated with 5 mM D-lactate for 6 h. bTNF-α was used as the postitive control. Each bar represents the mean ± SEM, *n* = 5. *p < 0.05; **p < 0.01.

To evaluate the direct proinflammatory role of D-lactate, we measured the expression of IL-6 in D-lactate-treated bFLS and detected a significant increase in the mRNA levels of this inflammatory gene at 6 h after stimulation (Figure 5A). bTNF-α, which was used as the positive control for pro-inflammatory cytokine expression, also significantly increased the mRNA levels of IL-6 (Figure 5A). Next, we evaluated the expression of HIF-1α, glucose transporter 1 (Glut-1), and pyruvate dehydrogenase kinase 1 (PDK-1). D-Lactate stimulation significantly increased the mRNA expression of HIF-1α, Glut-1, and PDK-1 in bFLS at 6 h after treatment (Figures 5B–D). Additionally, bTNF-α also increased the mRNA levels of these three metabolic genes (Figures 5B–D). Taken together, these results showed the ability of D-lactate to increase the expression of genes associated with both the inflammatory response and the cellular metabolism.

### D-Lactate Increases HIF-1α Protein Levels Under Normoxic Conditions in bFLS

**Figure 6:**
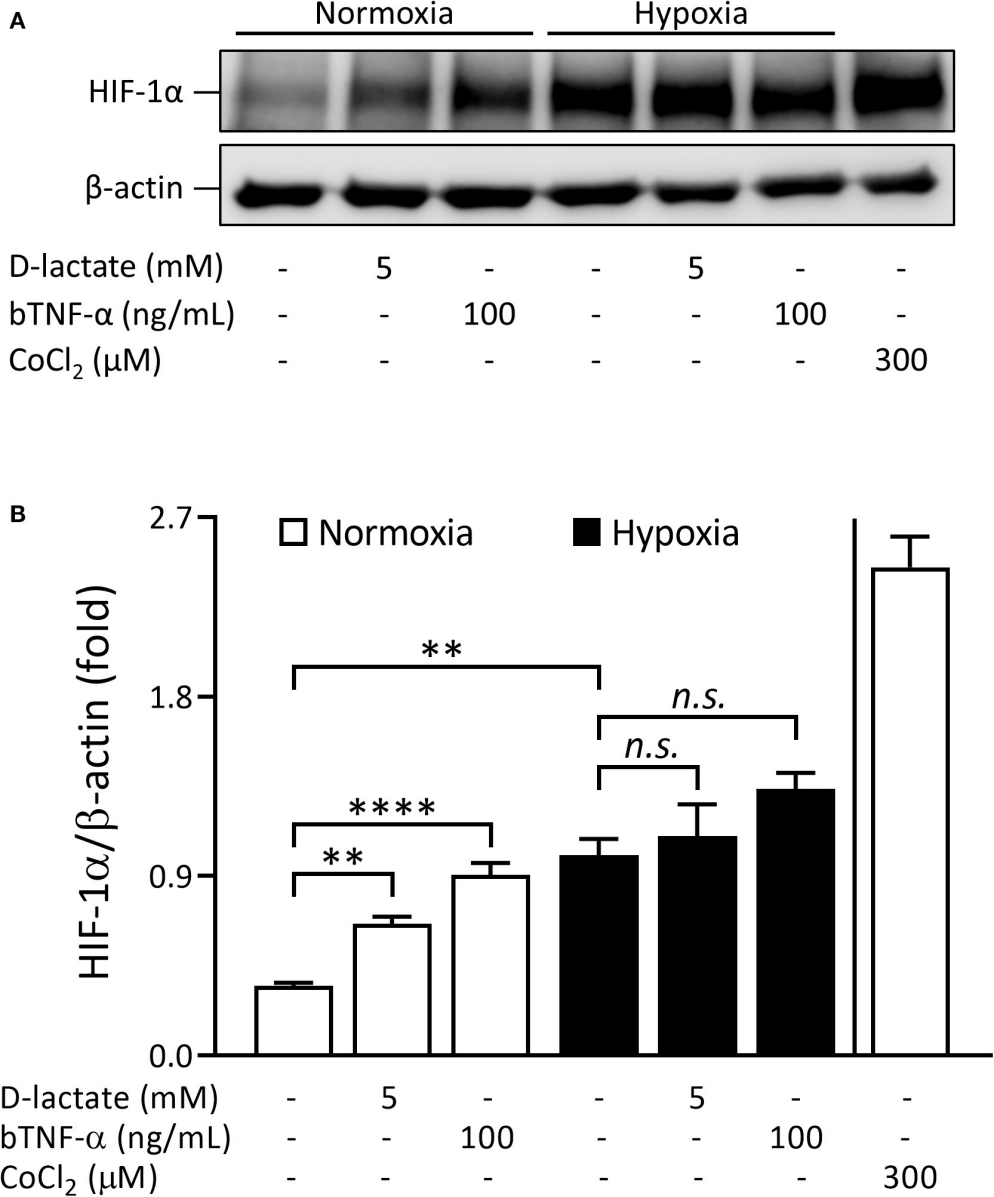
D-Lactate induces HIF-1α protein accumulation under normoxic conditions in bFLS. bFLS were stimulated with 5 mM D-lactate for 6 h under normoxic (20% O_2_) and hypoxic (1% O_2_) conditions. bTNF-α was used as the positive control. HIF-1α stabilization was detected in total protein extracts by Western blot using cobalt chloride (CoCl_2_) as the hypoxia-mimetic agent. **(A)** Representative HIF-1α immunoblot is shown. **(B)** Densitometry HIF-1α values were quantified using Image Studio Lite v5.2 software and normalized to β-actin. Each bar represents the mean ± SEM, *n* = 3. **p < 0.01; ****p < 0.0001; *n.s*., not significant.

Since D-lactate increased the mRNA expression of HIF-1α in bFLS, we also evaluated the ability of D-lactate to induce HIF-1α protein accumulation. Under normoxic conditions (20% O_2_), treatment of bFLS with D-lactate for 6 h significantly increased the HIF-1α protein levels compared to untreated cells (Figure 6). In addition, HIF-1α accumulation was also significantly higher in bTNF-α-treated bFLS relative to unstimulated control cells (Figure 6). Similar results were observed in bFLS exposed to D-lactate and bTNF-α under hypoxic conditions (1% O_2_), although the differences were not significant compared to the unstimulated cells in hypoxia (Figure 6). These results show the ability of D-lactate to induce HIF-1α protein accumulation in bFLS under normoxic conditions.

### D-Lactate-Induced IL-6, HIF-1α, Glut-1, and PDK-1 Expression in bFLS Is Dependent on the HIF-1 Activity

**Figure 7:**
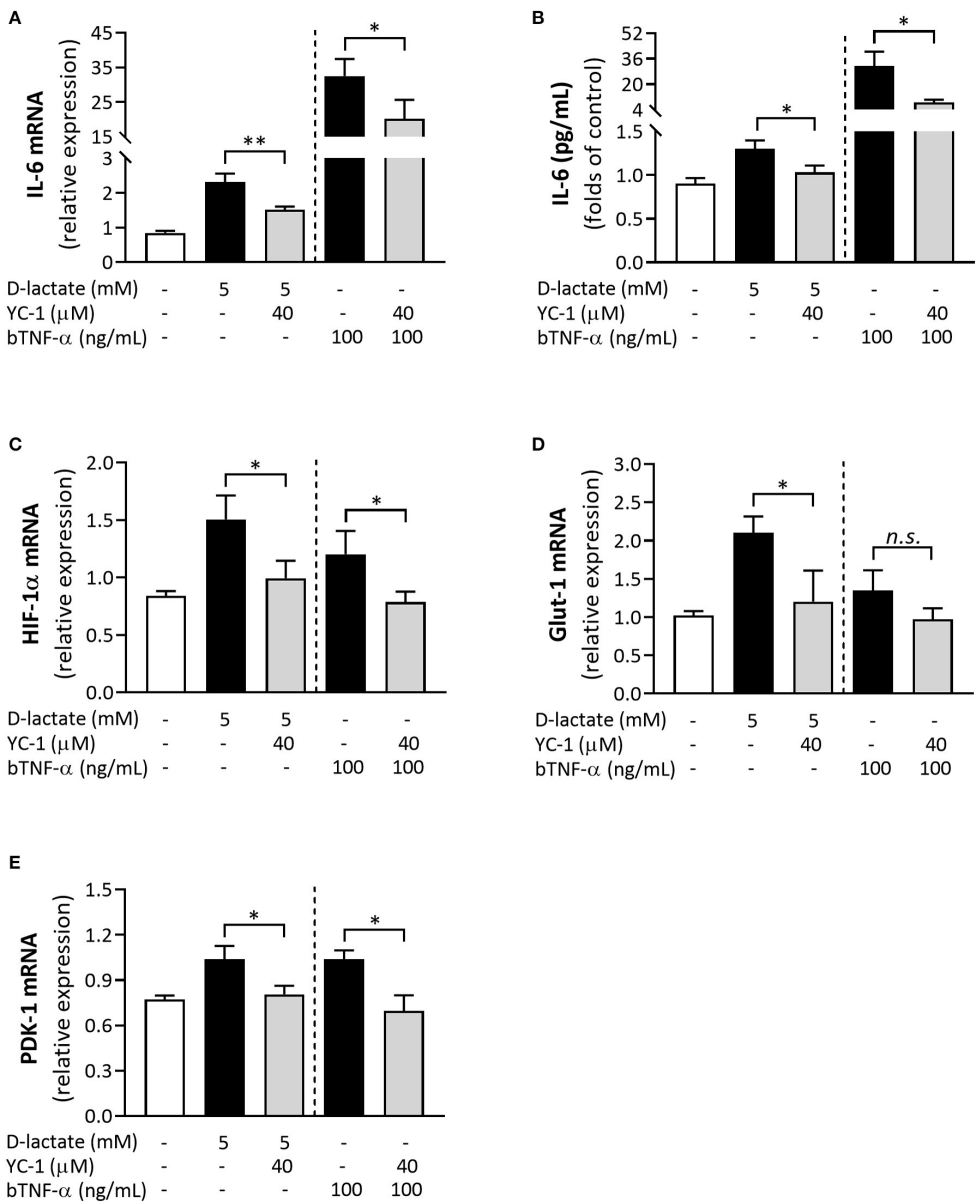
HIF-1 is involved in the D-lactate-induced overexpression of inflammation- and metabolism-associated genes in bFLS. bFLS were preincubated with 40 μM YC-1 and then stimulated with 5 mM D-lactate for 6 h. The relative mRNA expression levels of **(A)** IL-6, **(C)** HIF-1α, **(D)** Glut-1, and **(E)** PDK-1 are shown. **(B)** IL-6 levels in conditioned media were measured by ELISA. bTNF-α was used as the positive control. Each bar represents the mean ± SEM, *n* = 5. *p < 0.05; **p < 0.01; *n.s*., not significant.

The possible role of HIF-1 in the pro-inflammatory response induced by D-lactate in bFLS was evaluated by preincubating cells with YC-1, a synthetic compound with an inhibitory effect on HIF-1 activity (28, 29). Upon stimulation with D-lactate and bTNF-α, the mRNA levels of IL-6 were significantly lower in bFLS preincubated with YC-1 (Figure 7A). Similarly, the inhibition of HIF-1 also significantly decreased extracellular secretion of IL-6 induced by D-lactate and bTNF-α (Figure 7B). Since D-lactate and bTNF-α also increased the mRNA expression and secretion of IL-8 (23), we assessed the involvement of HIF-1 in this pro-inflammatory response. However, preincubation with YC-1 did not decrease the IL-8 expression or secretion (Supplementary Figure 3). We also evaluated the participation of HIF-1 in the mRNA expression of HIF-1α, Glut-1, PDK-1, and L-lactate dehydrogenase subunit A (L-LDHA). Preincubation of cells with YC-1 significantly reduced the mRNA levels of HIF-1α, Glut-1, PDK-1, and L-LDHA induced by D-lactate stimulation (Figures 7C–E; Supplementary Figure 4). In addition, YC-1 also significantly reduced the mRNA expression of HIF-1α and PDK-1 induced by bTNF-α treatment (Figures 7C,E). These results suggest that D-lactate-induced IL-6 mRNA expression and secretion are dependent on the HIF-1 activity. Similar to IL-6, D-lactate-mediated mRNA expression of HIF-1α, Glut-1, and PDK-1 was dependent on the HIF-1 activity.

### PI3K/Akt Signaling Pathway and the NF-κB Activity Mediate D-Lactate-Induced Expression of HIF-1α, Glut-1, and PDK-1 in bFLS

**Figure 8:**
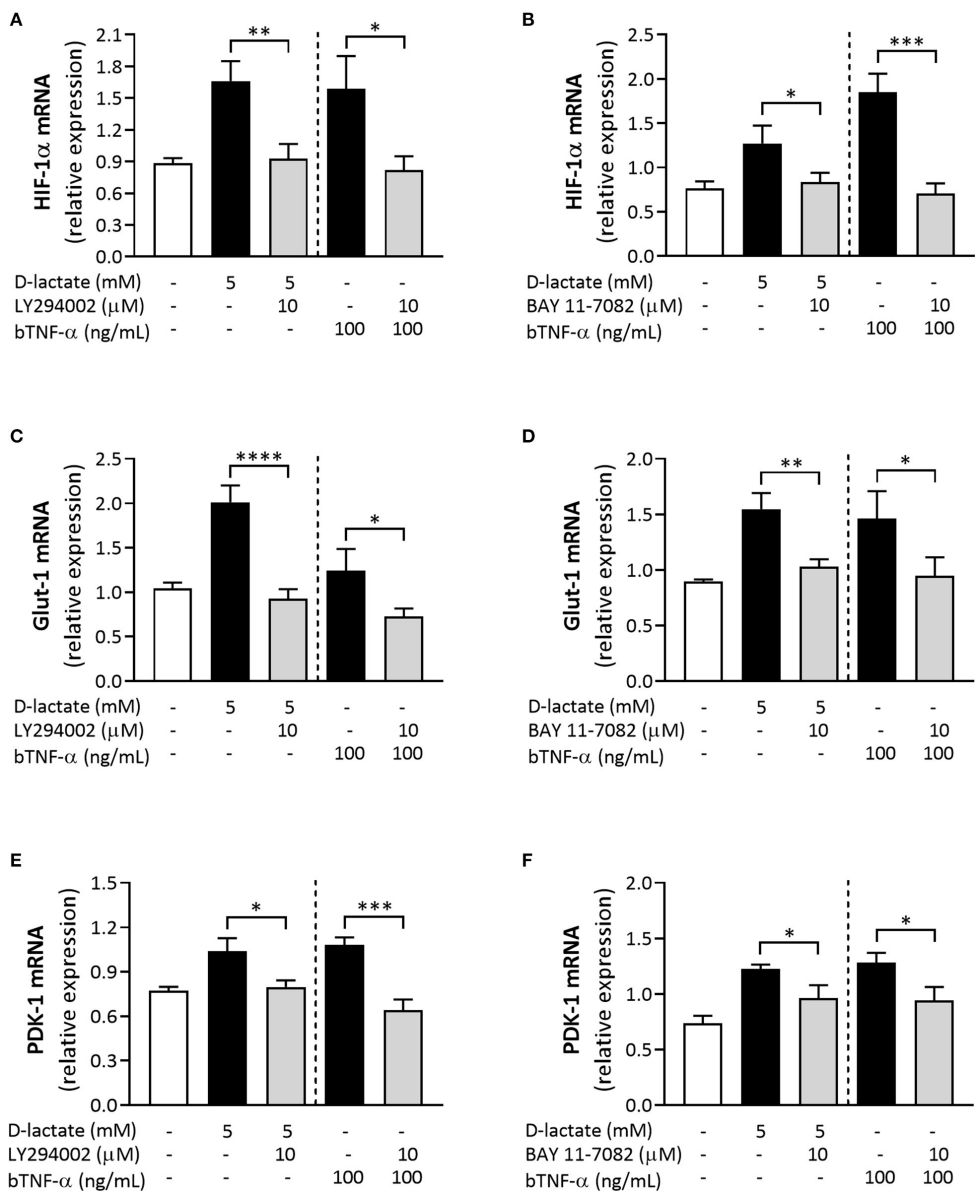
The PI3K/Akt pathway and the NF-κB pathway regulate the overexpression of metabolic genes induced by D-lactate in bFLS. bFLS were preincubated with 10 μM LY294002 or 10 μM BAY 11-7082 and stimulated with 5 mM D-lactate for 6 h. The relative mRNA expression levels of **(A,B)** HIF-1α, **(C,D)** Glut-1, and **(E,F)** PDK-1 are shown. bTNF-α was used as positive control. Each bar represents the mean ± SEM, *n* = 5. *p < 0.05; **p < 0.01; ***p < 0.001; ****p < 0.0001.

We used the pharmacological PI3K inhibitor LY294002 (30) to evaluate the involvement of the PI3K/Akt signaling axis in mRNA overexpression of metabolism-associated genes, such as HIF-1α, Glut-1, and PDK-1, induced by D-lactate and bTNF-α. Preincubation of cells with LY294002 significantly decreased the mRNA levels of HIF-1α (Figure 8A), Glut-1 (Figure 8C), and PDK-1 (Figure 8E) induced by both D-lactate and bTNF-α. Finally, we also used BAY 11-7082, a synthetic inhibitor of IκB-α phosphorylation (31), to evaluate the involvement of NF-κB activity in the altered mRNA expression of HIF-1α, Glut-1, and PDK-1 induced by D-lactate and bTNF-α. Inhibition of NF-κB activity also significantly decreased the mRNA expression of HIF-1α (Figure 8B), Glut-1 (Figure 8D), and PDK-1 (Figure 8F) induced by both D-lactate and bTNF-α. Taken together, these results suggest that D-lactate induces the expression of metabolic genes, namely HIF-1α, Glut-1, and PDK-1, *via* PI3K/Akt-dependency, as well as the NF-κB activity, in exposed bFLS.

## Discussion

Lameness and its adverse implications for animal welfare and health have become recognized as problems in recent years, particularly in the intensive dairy cattle farming industry (32, 33). High energy diets rich in easily available carbohydrates favor acidotic states, the consequences of which include the occurrence of laminitis, a diffuse, aseptic inflammation of the corium, and aseptic polysynovitis, which also contributes to lameness (3, 9). In addition, neutrophil recruitment in the joints of heifers with ARA has been reported, although its pathophysiology has yet to be studied in more detail (9, 10). Alarcon et al. reported high concentrations of D-lactate (~5 mM) and metabolic disturbances in the synovial fluid extracted from heifers with ARA (17). Given the central role of bFLS in maintaining the synovial fluid composition and contributing to inflammatory and metabolic changes during joint diseases (14), we first evaluated the effect of D-lactate on bFLS metabolome.

Concentrations of several carbohydrates were significantly higher in bFLS exposed to 5 mM D-lactate than that observed in untreated cells. Based on these metabolites, primary metabolic pathways altered after D-lactate treatment were “glycolysis/gluconeogenesis” as well as “galactose metabolism.” GC-MS metabolomic profiling of the synovial fluid of heifers with ARA at 9 h post oligofructose overload primarily included the “starch and sucrose metabolism,” “galactose metabolism,” and “glycolysis or gluconeogenesis” pathways (17). Comparative metabolomic analysis of cultured FLS from patients with rheumatoid arthritis (RA) and osteoarthritis (OA) showed that the altered primary metabolic pathways were “glycolysis and gluconeogenesis,” “galactose metabolism,” and “fructose and mannose metabolism” (16). Supporting our results, disturbances of carbohydrate metabolism appear to be key in the aseptic inflammatory joint (34).

The levels of several amino acids, including threonine, were significantly increased by D-lactate treatment. Threonine is one of the most abundant amino acids present in the bovine synovial proteins and fluid (35, 36), and its augmented levels have been observed in the joints of patients with OA (16, 37). Threonine is considered to be a glucogenic amino acid that can be converted into pyruvic acid for energy supply in organisms (38, 39). In addition, its putative role in glucose and pyruvate metabolism during the inflammatory response has also been previously reported (16, 40).

A significant increase in the level of several saturated long-chain fatty acids was observed in bFLS treated with D-lactate. Linoleic acid, an omega-6 polyunsaturated fatty acid, was also higher in stimulated cells. Interestingly, increased levels of fatty acids were also detected in the synovial fluid of heifers with ARA at 24 h after oligofructose overload (17), suggesting that lipid metabolism may be a metabolic pathway involved during inflammatory processes in synovial cells. Supporting the above hypothesis, Ahn et al. suggested that significantly higher levels of fatty acids in RA-FLS than OA-FLS were due to increased lipolysis in inflamed tissues for energy production (16). Additionally, omega-6 polyunsaturated fatty acids are precursors to several pro-inflammatory eicosanoids and prostaglandins through the arachidonic acid cascade, which actively participates in the pro-inflammatory process (41). Furthermore, Hidalgo et al. reported high levels of PGE_2_ in the synovial fluid of heifers at 9 and 24 h after oligofructose overload (42).

In the present study, increased levels of glucose, pyruvic acid, succinic acid, and fumaric acid were detected by GC-MS in bFLS stimulated with D-lactate. Additionally, we also detected a significant increase in the intracellular levels of L-lactate in stimulated bFLS by HPLC analysis together with an intracellular accumulation of D-lactate, which was attributable to transport mechanisms *via* monocarboxylate transporter 1 (43). These metabolites were associated with alterations in “pyruvate metabolism” and “citrate cycle (TCA cycle).” According to our results, high levels of lactate have been detected in the synovial fluid of patients with aseptic inflammatory conditions, such as RA (44–46) and gouty (47). In addition, Borenstein et al. also reported higher levels of lactic, fumaric, and succinic acids in non-septic inflammatory synovial fluids than in non-inflammatory fluids (48). Alarcon et al. also reported higher levels of D-lactate, L-lactate, and pyruvic acid in the synovial fluid of heifers with ARA at 9 h after oligofructose overload, which was associated with an upregulation of the “pyruvate metabolism” and “glycolysis or gluconeogenesis” pathways (17). Thus, our results demonstrate the ability of D-lactate to induce metabolic reprogramming in bFLS and support the hypothesis that it has a central role in the metabolic changes detected in the synovial fluid of heifers with ARA.

During inflammation, cells need to generate energy and biomolecules to support growth, proliferation, and pro-inflammatory molecule production, resulting in cell metabolism shifts toward glycolysis (49, 50). Moreover, a metabolic shift toward anaerobic glycolysis enables cells to better cope with metabolically restrictive conditions during inflammation, such as those that occur in the transition from normoxia to hypoxia (49). FLS from patients with RA have increased glycolytic activities characterized by an elevated expression of glycolytic markers, such as hexokinase 2 and Glut-1 (51). In these cells, a metabolic switch to anaerobic glycolysis is essential to support angiogenesis, cellular invasion, and pannus formation (52). Indeed, glycolysis blockade has been shown to ameliorate inflammation and subsequent cartilage damage in several models of arthritis (51, 53). Similarly, studies on macrophages and dendritic cells focusing on metabolic adaptations have highlighted the key role of glycolysis in the initiation and development of inflammation induced by danger signals (54).

Supporting the above, D-lactate treatment increased the mRNA expression of IL-6 in bFLS, a response also observed in bFLS treated with bTNF-α. IL-6 is a key pro-inflammatory cytokine in numerous joint diseases (55, 56). Indeed, the expression of IL-6 and the severity of lesions in aseptic joint diseases are correlated (55, 57). Furthermore, IL-6 levels were shown to be increased in the synovial fluid of heifers with ARA at 9 and 24 h after oligofructose overload (17, 42), and incubating bFLS with 2 and 5 mM D-lactate significantly increased the mRNA expression of IL-6 (17).

HIF-1 is a master regulator of the transcription of hundreds of genes required to maintain a balance between oxygen supply and metabolic demand (58). HIF-1 is a heterodimeric protein complex comprising an oxygen-sensitive α subunit (HIF-1α), which is degraded by the proteasomal pathway under normoxic conditions, and an oxygen-insensitive β subunit (HIF-1β) (59, 60). Under hypoxic conditions, accumulation of HIF-1α induces HIF-1-regulated adaptive responses that facilitate the production of glycolytic ATP, including the transcription of Glut-1, hexokinase (HK), PDK-1, and enzymes of the glycolytic pathway (61–63). In the present study, treatments with D-lactate and bTNF-α induced the accumulation of HIF-1α protein under normoxic conditions in bFLS. Consistent with our findings, HIF-1 activation under normoxic conditions has also been reported after cellular exposure to pro-inflammatory agents, including growth factors; bacteria; and their compounds, namely TNF-α, IL-1β, and lactate, among other agents (20, 21, 64–68). In addition, D-lactate treatment also increased the mRNA expression of HIF-1α, Glut-1, PDK-1, and L-LDHA in stimulated bFLS. To assess the role of HIF-1 in this response, we used the HIF-1-inhibitor YC-1, which enhances the binding of factor inhibitor of HIF-1 (FIH) to the transactivation domain COOH-terminal (C-TAD) in the HIF-1α subunit, dissociating the binding of the latter to the p300 coactivator and leading to the functional repression of HIF-1 (29). YC-1 significantly reduced the gene expression of HIF-1α, Glut-1, PDK-1, and L-LDHA, suggesting the involvement of HIF-1 in the metabolic reprogramming induced by D-lactate in bFLS under normoxic conditions. Similarly, YC-1 also significantly decreased the gene expression of bTNF-α-induced HIF-1α, PDK-1, and Glut-1. Consistent with the results, previous reports also showed the involvement of HIF-1 in the mRNA expression of HIF-1α (69), Glut-1 (70), and PDK-1 (62). Interestingly, YC-1 also decreased the IL-6 mRNA expression, suggesting the involvement of HIF-1 in the upregulation of gene expression of this relevant pro-inflammatory cytokine induced by D-lactate and bTNF-α in bFLS. Based on the results, findings of other related studies also showed an increased production of IL-6 through a HIF-1-dependent mechanism in synoviocytes and chondrocytes using an experimental model of ischemic osteonecrosis (56), while the ability of lactate to induce IL-6 secretion through a HIF-1-dependent pathway was also reported by other researchers in chondrocytes (20). In contrast, in bFLS, D-lactate and bTNF-α induced the expression and secretion of IL-8 in a HIF-1-independent manner, suggesting a selective role of HIF-1 in the expression of pro-inflammatory genes in synovial cells. In support of this, HIF-1α knock-down in RA-FLS did not reduce the expression of IL-8 and MMP-1 induced by hypoxia (71).

The PI3K/Akt pathway has a central role in the regulation of cell growth and metabolism in different host cell types (72). Consequently, Akt regulates several processes associated with glucose metabolism, including Glut-1 localization to the cell membrane, pentose phosphate shuttle activity, and the activation of various glycolytic enzymes such as HK and phosphofructokinase (73, 74). We recently described that D-lactate induced Akt phosphorylation, and inhibition of the PI3K/Akt pathway reduced the mRNA expression and secretion of IL-6 and IL-8 (23). In the present study, PI3K/Akt pathway inhibition by LY294002 (30) significantly reduced the mRNA levels of IL-6, HIF-1α, Glut-1, and PDK-1 induced by D-lactate and bTNF-α. Similarly, the PI3K inhibitor LY294002 interfered with TNF-α-induced activation of OA-FLS, attenuating the overexpression of cadherin-11 and reducing the invasive ability of these cells (75). Similarly, Jia et al. demonstrated that the PI3K/Akt pathway inhibition by LY294002 or cucurbitacin E significantly reduced the TNF-α-induced production of IL-1β, IL-6, and IL-8 mRNA and the protein expression in human synoviocytes (76). A dependence on the PI3K/Akt pathway for the expression of pro-inflammatory cytokines, namely IL-6, IL-8, IL-17a, and IL-1β, was also reported by Li et al. in synovial fibroblasts isolated from rats with experimental OA (77). Similarly, in human tumoral cell lines, pharmacological inhibition of the PI3K/Akt pathway was shown to reduce the mRNA expression of Glut-1 (78–80) as well as the protein levels of HIF-1α and PDK-1 (81).

The results of previous studies suggest that the PI3K/Akt pathway regulates the nuclear translocation of NF-κB (76, 82). Thus, to evaluate the role of NF-κB in inflammatory and metabolic responses induced by D-lactate, we preincubated bFLS with the pharmacological inhibitor BAY 11-7082, which inhibits IκB-α phosphorylation and interferes with NF-κB nuclear translocation, functionally inactivating the pathway (31). The results of the study demonstrated that inactivation of the NF-κB pathway strongly decreased mRNA levels of IL-6 induced by D-lactate and bTNF-α in exposed bFLS. Recently, we demonstrated that D-lactate and bTNF-α induced the degradation of IκBα after 30 min of stimulation in bFLS, and treatment with BAY 11-7082 significantly reduced the expression and secretion of IL-6 and IL-8 induced by both stimuli, supporting that the NF-κB pathway is induced by D-lactate in bFLS (23). The NF-κB activity was also shown to be key for the expression of several pro-inflammatory cytokines in bovines, including IL-6, TNF-α, and IL-1β (83–87). Similarly, the NF-κB activity is strongly involved in the mRNA expression of IL-6, IL-8, and IL-1β induced by TNF-α in human FLS (82, 88, 89). Interestingly, the gene expression of HIF-1α, Glut-1, and PDK-1 induced by D-lactate and bTNF-α was also significantly inhibited by BAY 11-7082. The role of NF-κB in cell metabolic reprogramming has been poorly investigated, although it was reported that the RelA subunit of NF-κB upregulates the transcription of Glut-3, increasing glucose uptake and glycolytic flux (90), as well as upregulates mitochondrial respiration (91) in murine primary cultured cells. Furthermore, the role of the NF-kB pathway in HIF-1 mRNA expression in RA-FLS stimulated with IL-17A has also been demonstrated (92), suggesting its participation in metabolic reprogramming during inflammation.

Nonetheless, although we demonstrated the role of HIF-1 in metabolic and inflammatory responses induced by D-lactate in FLS, we cannot rule out the involvement of other upstream signaling pathways that could be activated by D-lactate, thus regulating HIF-1 activity. Several studies have described involvement of extracellular-regulated kinase (ERK), PI3K/Akt, and mammalian target of rapamycin (mTOR) pathways in the regulation of HIF-1α mRNA and protein levels after stimulation with proinflammatory cytokines and growth factors (64, 93–99). Therefore, additional studies are required to elucidate precise mechanisms involved in HIF-1 activation induced by D-lactate in bFLS of joints.

**Figure 9:**
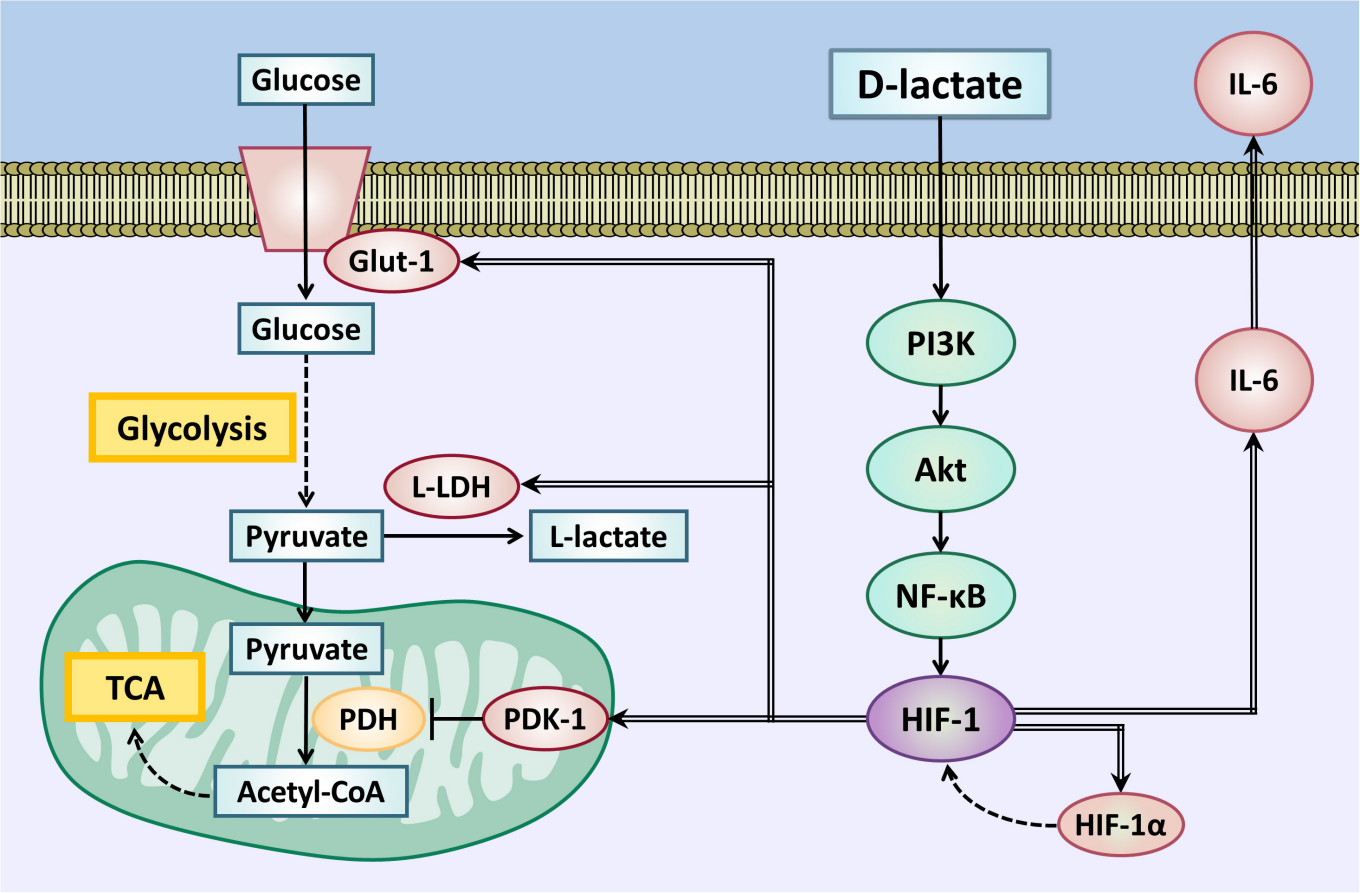
Metabolic reprogramming supports the inflammatory response induced by D-lactate in bFLS. D-Lactate induces the PI3K/Akt pathway activation and downstream activation of NF-κB and HIF-1. Through this signaling pathway, D-lactate induces the gene expression of IL-6, with the pro-inflammatory cytokine involved in the synovial inflammatory response. HIF-1 activation also increases the expression of the Glut-1 transporter, which increases glucose uptake for use in glycolysis. Glycolysis is also favored by the HIF-1-dependent overexpression of L-LDH, which oxidizes pyruvate to L-lactate. In addition, an increased PDK-1 expression blocks the mitochondrial utilization of pyruvate through the TCA cycle, contributing to the glycolytic fate of glucose. Overexpression of the HIF-1α subunit would favor the accumulation of HIF-1 heterodimers, maintaining glycolytic metabolic reprogramming. IL-6, interleukin 6; Glut-1, solute carrier family 2 (facilitated glucose transporter) member 1; L-LDH, L-lactate dehydrogenase; PDK-1, pyruvate dehydrogenase kinase 1; HIF-1α, hypoxia inducible factor 1 subunit alpha; PDH, pyruvate dehydrogenase; TCA, tricarboxylic acid cycle; PI3K, phosphatidyl inositol 3-kinase; Akt, protein kinase B; NF-κB, nuclear factor kappa B; HIF-1, hypoxia-inducible factor 1.

In conclusion, D-lactate induces an inflammatory response along with metabolic reprogramming in bFLS. Both processes are dependent on activities of transcription factors, such as HIF-1 and NF-κB, as well as the activation of the PI3K/Akt signaling pathway, which contribute to an increased expression of IL-6, HIF-1α, Glut-1, PDK-1, and L-LDHA (Figure 9). These results support the pivotal role of D-lactate in bovine joint inflammation and glycolytic metabolic disturbances observed in synovitis induced by ARA in cattle.

## Data Availability Statement

The original contributions presented in the study are included in the article/Supplementary Material, further inquiries can be directed to the corresponding author/s.

## Ethics Statement

The animal study was reviewed and approved by Ethical committee of Universidad Austral de Chile #0023/18; Valdivia, Chile.

## Author Contributions

JQ, PA, MC, and RB designed the experiments. JQ, PA, CM, and ST performed the experiments. JQ, PA, and RB prepared the manuscript. AT, CH, MH, and RB analyzed the data. All authors have read and approved the final version of this manuscript.

## Conflict of Interest

The authors declare that the research was conducted in the absence of any commercial or financial relationships that could be construed as a potential conflict of interest.
