# Supplementary material for: Metabolic Reprogramming and Inflammatory Response Induced by D-Lactate in Bovine Fibroblast-Like Synoviocytes Depends on HIF-1 Activity
Source: Front Vet Sci. 2021 Mar 16;8:625347. doi: 10.3389/fvets.2021.625347 (PMC8007789; doi:10.3389/fvets.2021.625347)
Supplement: Supplementary file 1 [file Data_Sheet_1.docx]

Supplementary Material


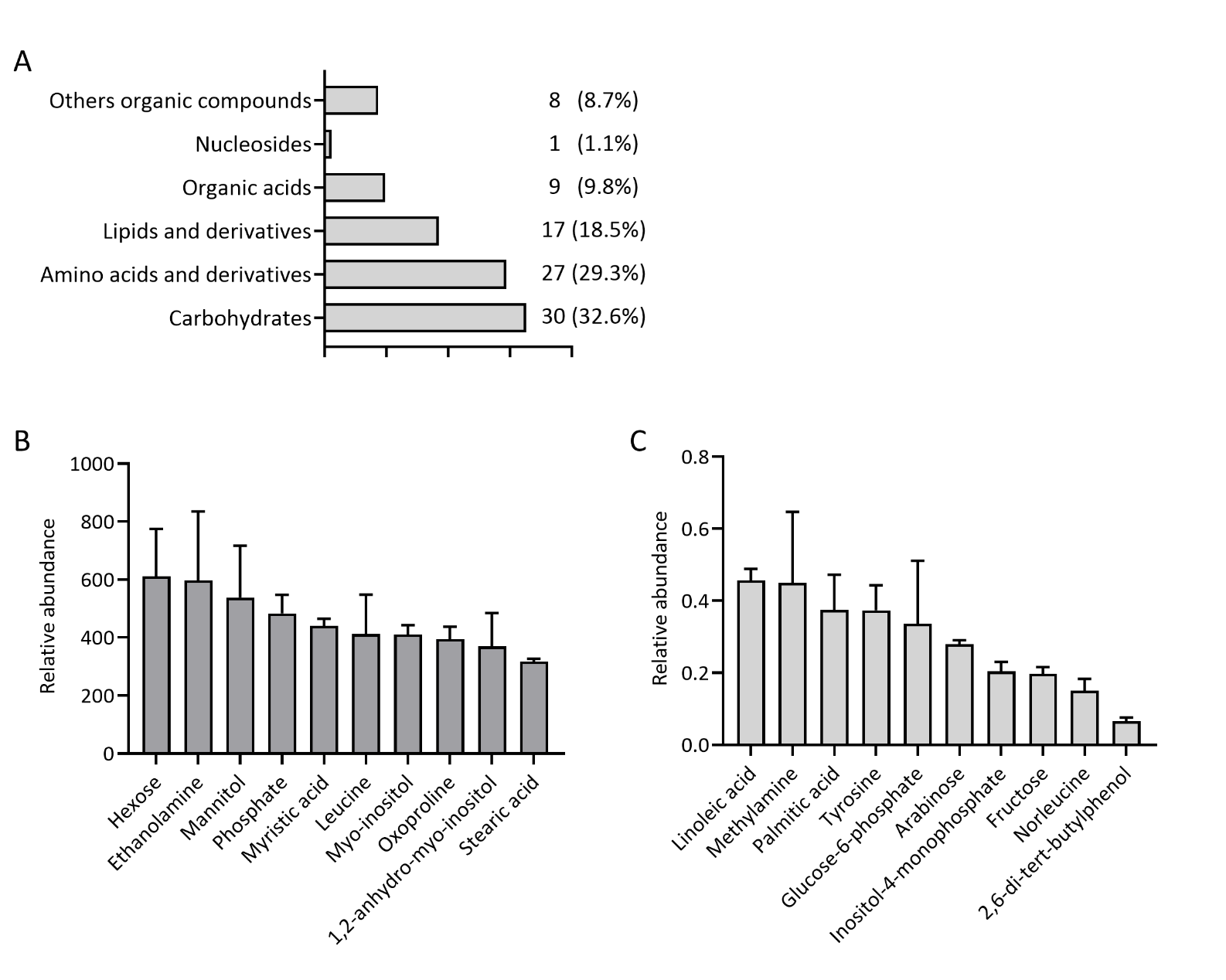


**Supplementary Figure 1. Metabolomic profile of bFLS before D-lactate stimulation.** **(A)** Classification of 93 metabolites detected by gas chromatography-mass spectrometry (GC-MS) in bFLS according to chemical class. Each bar represents the relative abundance of the chemical class. The absolute and relative abundance (%) of each class is also indicated. **(B)** The 10 most abundant metabolites identified in bFLS by GC-MS. **(C)** The 10 metabolites with the lowest abundance detected in bFLS by GC-MS. Each bar represents the mean ± SEM, n = 4.


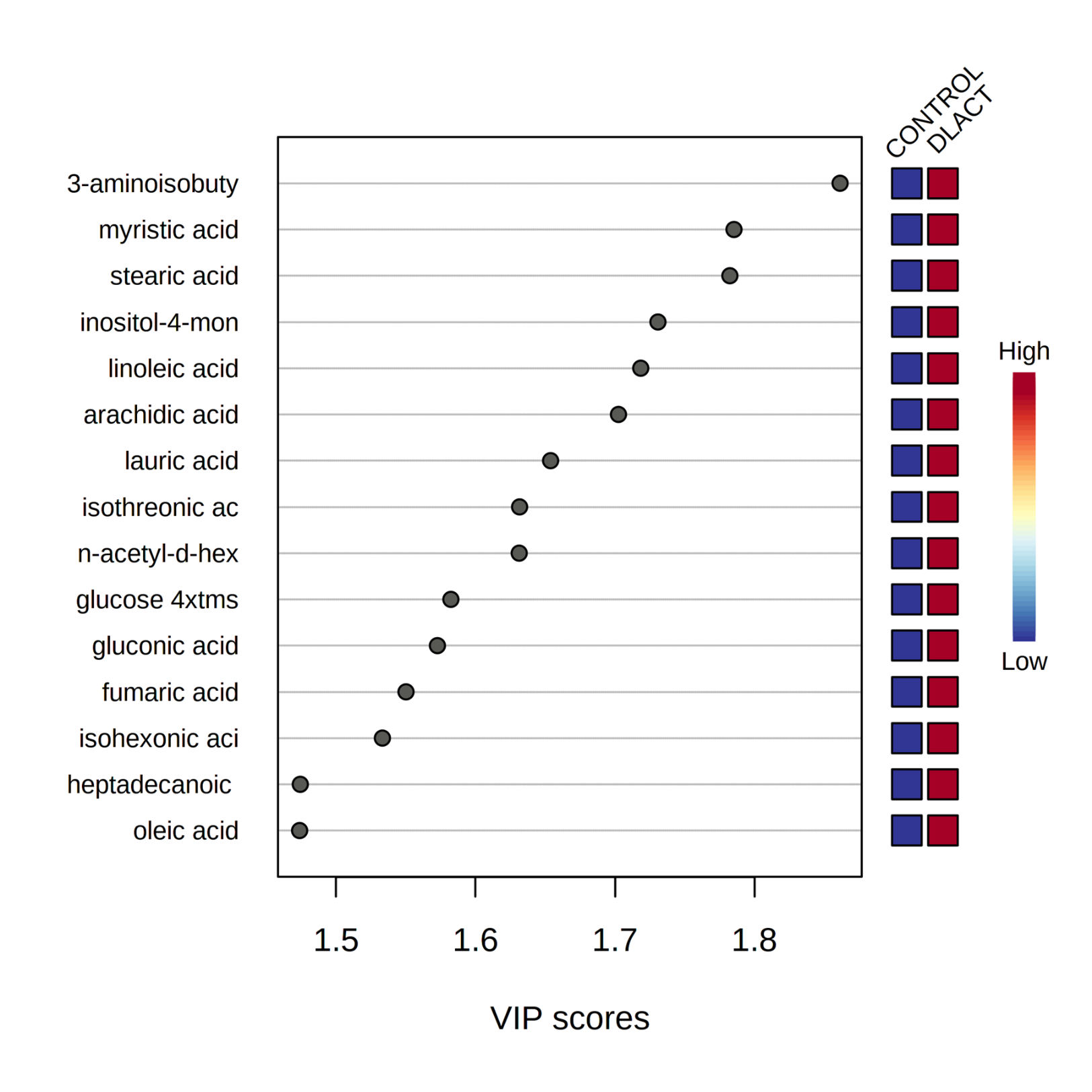


**Supplementary Figure 2. Variable importance in projection (VIP) scores** **of primary metabolites identified by partial least squares-discriminant analysis (PLS-DA).** The 15 metabolites with the highest VIP scores are shown. Colored boxes on the right represent the relative concentrations of metabolites in each group. The red and blue colors indicate that the metabolite level is increased or decreased with respect to the mean of the relative metabolite abundance. n = 4.


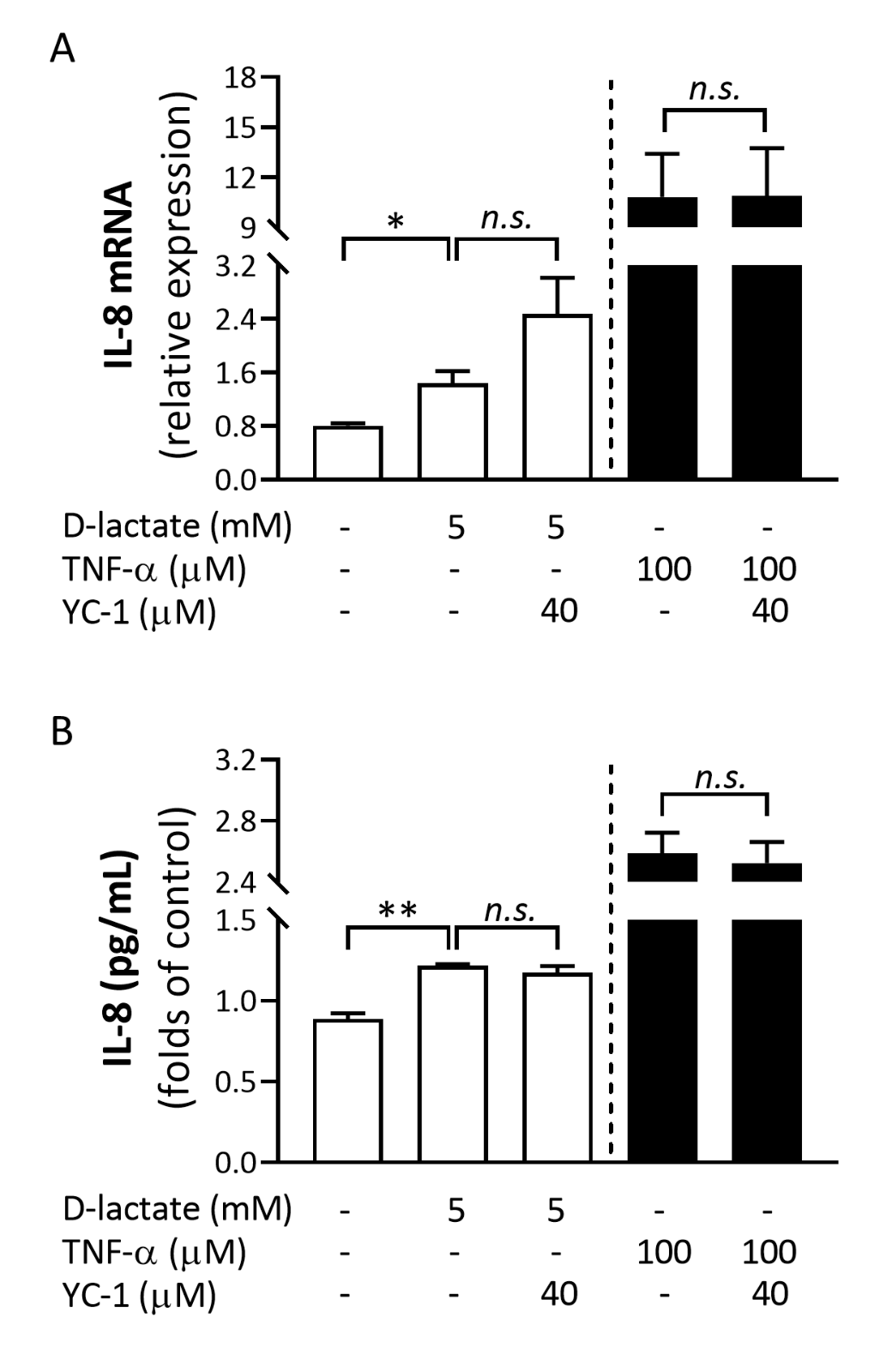


**Supplementary Figure 3.** **D-lactate increases the overexpression of IL-8 independently of HIF-1 activity.** bFLS were preincubated with 40 μM YC-1 and then stimulated with 5 mM D-lactate for 6 h. bTNF-α was used as positive control. (A) The relative mRNA expression of IL-8 is shown. (B) IL-8 levels in conditioned media were measured by ELISA. Each bar represents the mean ± SEM, n = 5. * = *p* < 0.05; ** = *p* < 0.01; *n.s.* = not significant.


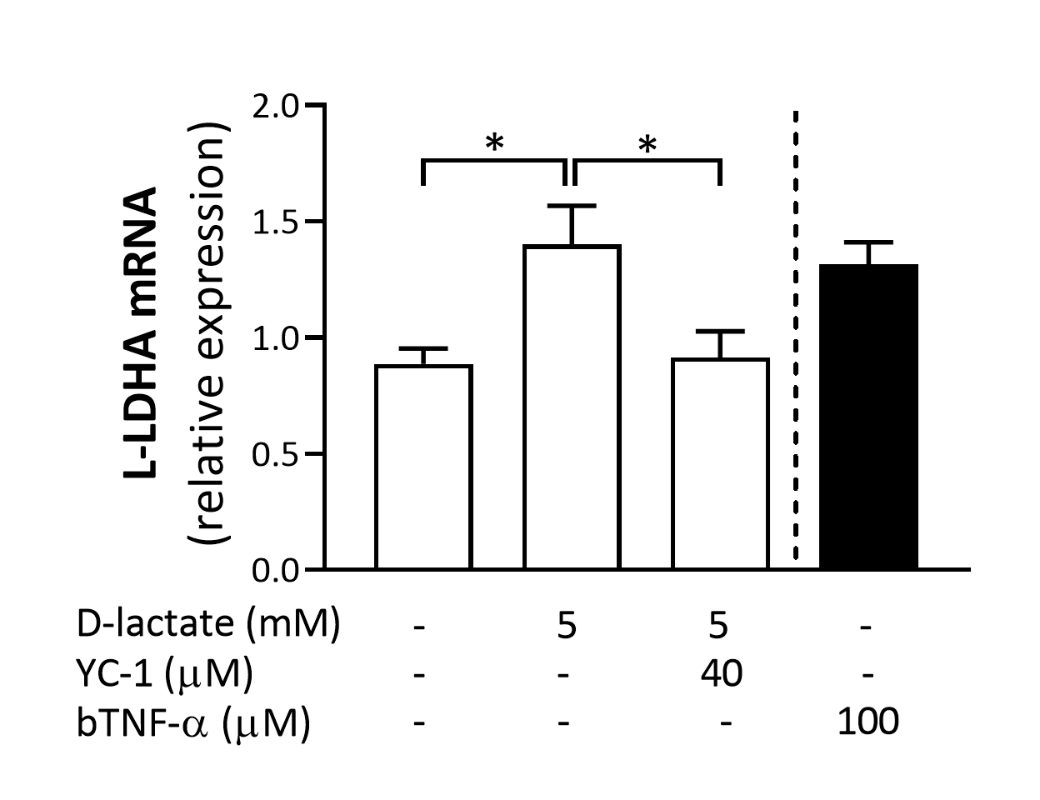


**Supplementary Figure 4.** **HIF-1 is involved in the overexpression of L-LDHA induced by D-lactate in bFLS.** bFLS were preincubated with 40 μM YC-1 and then stimulated with 5 mM D-lactate for 6 h. The relative mRNA expression of L-LDHA is shown. bTNF-α was used as positive control. Each bar represents the mean ± SEM, n = 5. * = *p* < 0.05.
